# Gene expression in normal-appearing tissue adjacent to prostate cancers are predictive of clinical outcome: evidence for a biologically meaningful field effect

**DOI:** 10.18632/oncotarget.8944

**Published:** 2016-04-22

**Authors:** Cristina Magi-Galluzzi, Tara Maddala, Sara Moscovita Falzarano, Diana B. Cherbavaz, Nan Zhang, Dejan Knezevic, Phillip G. Febbo, Mark Lee, Hugh Jeffrey Lawrence, Eric A. Klein

**Affiliations:** ^1^ Pathology and Laboratory Medicine Institute, Cleveland Clinic, Cleveland, Ohio, USA; ^2^ Glickman Urological and Kidney Institute, Cleveland Clinic, Cleveland, Ohio, USA; ^3^ Genomic Health, Inc., Redwood City, California, USA

**Keywords:** prostate cancer, gene expression profiling, molecular diagnostics, prognosis, risk assessment

## Abstract

**Purpose:**

We evaluated gene expression in histologically normal-appearing tissue (NT) adjacent to prostate tumor in radical prostatectomy specimens, assessing for biological significance based on prediction of clinical recurrence (cR - metastatic disease or local recurrence).

**Results:**

A total of 410 evaluable patients had paired tumor and NT. Fortysix genes, representing diverse biological pathways (androgen signaling, stromal response, stress response, cellular organization, proliferation, cell adhesion, and chromatin remodeling) were associated with cR in NT (FDR < 20%), of which 39 concordantly predicted cR in tumor (FDR < 20%). Overall GPS and its stromal response and androgen-signaling gene group components also significantly predicted time to cR in NT (RM-corrected HR/20 units = 1.25; 95% CI: 1.01-1.56; P = 0.024).

**Experimental Design:**

Expression of 732 genes was measured by quantitative reverse transcriptase polymerase chain reaction (RT-PCR) separately in tumor and adjacent NT specimens from 127 patients with and 374 without cR following radical prostatectomy for T1/T2 prostate cancer. A 17-gene expression signature (Genomic Prostate Score [GPS]), previously validated to predict aggressive prostate cancer when measured in tumor tissue, was also assessed using pre-specified genes and algorithms. Analysis used Cox proportional hazards models, Storey's false discovery rate (FDR) control, and regression to the mean (RM) correction.

**Conclusions:**

Gene expression profiles, including GPS, from NT adjacent to tumor can predict prostate cancer outcome. These findings suggest that there is a biologically significant field effect in primary prostate cancer that is a marker for aggressive disease.

## INTRODUCTION

There is a clear medical need for prostate cancer (PCa) biomarkers that provide accurate risk stratification to inform clinical decision making for men with newly diagnosed disease. The intrinsic heterogeneity and multifocal nature of PCa have challenged the development of robust molecular markers. One approach to addressing these challenges is to incorporate a deeper understanding of the biology of the non-malignant cells present within and adjacent to the tumor, which may represent a generalized field effect related to tumor aggressiveness. The concept of field cancerization has evolved from a histological definition first credited to Slaughter et al. in 1953 [1] to a molecular definition exemplified by a range of genetic and epigenetic abnormalities that can be detected in normal-appearing tissues (NT) adjacent to cancers, including gene silencing by methylation [2], deletions in mitochondrial DNA [3], mutations in cancer-related genes [4], and aberrant gene expression [5, 6]. Evidence for a field effect has been reported in a variety of cancer types, including head and neck [7], lung [8], colon [9], breast [10], stomach [11], bladder [12] and PCa [13, 14].

We undertook a series of studies to develop a tumor-based gene expression signature that would provide a biologic measure of tumor aggressiveness and improve risk assessment for men with newly diagnosed, clinically low-risk PCa. As previously described [15], we screened the expression of 732 cancer-related genes in radical prostatectomy (RP) tumor specimens from 441 patients with clinically localized disease, and identified genes whose expression was predictive of clinical outcome. To create an assay that was a robust predictor of outcome in the face of heterogeneity in grade and multifocality, we selected genes that were predictive of clinical recurrence (metastases or locoregional recurrence) in two separate regions of each tumor. From those studies, we identified a final set of 12 cancer-related genes, representing several biological pathways, and 5 reference genes which were combined in an algorithm to generate a 100-point Genomic Prostate Score (GPS). The clinical-grade assay has been analytically [16] and clinically validated to predict the likelihood of adverse pathology at radical prostatectomy and risk of biochemical recurrence when performed on RNA from fixed paraffin-embedded prostate needle biopsy specimens [15, 17]. The assay has been shown to influence clinical decision making by distinguishing men with biologically non-aggressive tumors who are appropriate candidates for active surveillance from men with clinically low risk disease who have biologically aggressive tumors and should be considered for immediate therapy [18].

A pre-specified aim of the original gene identification study conducted on RP specimens was to assess gene expression in NT adjacent to prostate tumor, where “adjacent” was defined as ≥3-mm distant from tumor. The goal of this analysis was to identify gene expression changes in NT that could predict clinical outcome and to compare the gene expression patterns from NT to tumor tissue. Additionally, post-hoc analyses were conducted to evaluate if GPS, developed using the tumor-containing tissue, was predictive of outcome when applied to gene expression derived from NT specimens. In this study we asked whether identification of genes in NT would provide evidence for a biologically meaningful field effect in the tumor-containing prostate and identify early markers of aggressive PCa assayable in non-tumor tissue.

## RESULTS

### Patient characteristics

**Figure 1:**
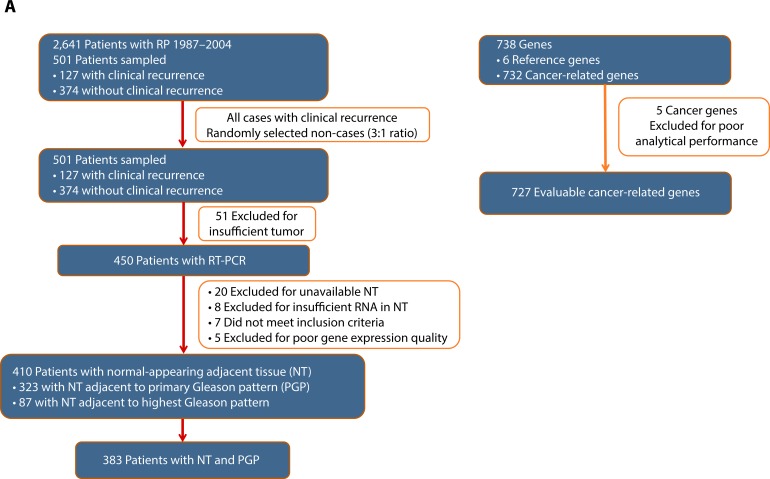

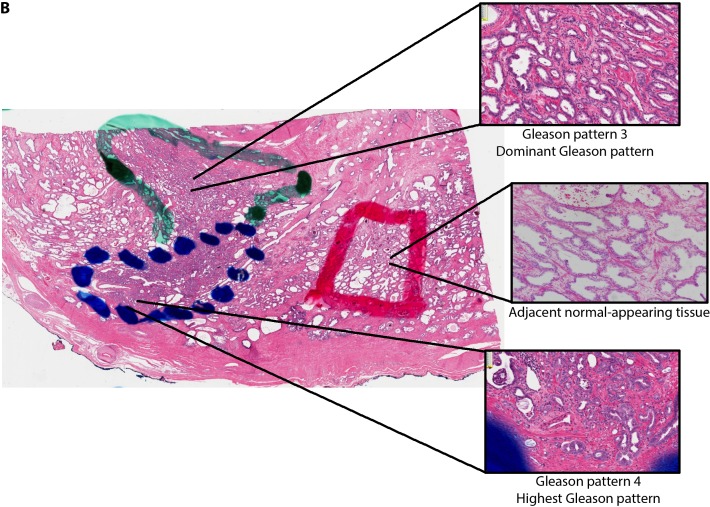
Panel **a.** Evaluable Patients, Samples and Genes. A total of 2,641 patients were identified for the study; 501 patients were sampled to include all clinical recurrences (127) and 3 times as many non-recurrences (374) and were representative of the full identified cohort. A total of 91 patients were excluded due to unevaluable tissue (tumor or NT) or were clinically ineligible resulting in 410 evaluable patients. A total of 732 cancer-related genes were measured and 5 were excluded due to poor analytical performance. Six reference genes were evaluated. Panel **b.** Tumor and NT Specimens.

Of the 501 radical prostatectomy specimens selected for the original gene identification study [15], 410 patients were evaluable for this study. Exclusions included insufficient tumor for analysis (51 cases), lack of available NT tissue or insufficient RNA in NT (28 cases), clinical exclusions (7 cases), and poor RNA expression quality (5 cases) (Figure 1a). Among these patients, 383 had gene expression data required to calculate GPS from paired NT and tumor specimens. The majority (*n* = 323 with weighted percentage of 80%) of patients had NT sampled adjacent to their primary Gleason pattern specimen. Patients were mostly Caucasian (84%), had a median age of 61 years, and presented with AUA-low (57%) or intermediate-risk (33%) disease (Table 1). The majority of patients had multifocal tumors (77%). The median follow-up time was 5.6 years, and included 93 clinical recurrences. The distribution of baseline characteristics for the 383 patients used in this analysis was representative of the full database of 2,641 patients (Table 1). As expected, in univariate models, preoperative PSA, clinical T-stage, biopsy Gleason score, pathologic T-stage, surgical Gleason score, year of surgery, surgical margin status, and AUA risk group were all significantly (*P* < 0.05) associated with clinical recurrence (data not shown).

**Table 1:** Baseline characteristics of patient population

Characteristic		All Evaluable Patients in Study (*N* = 410)	All Patients in CC Cohort (*N* = 2,641)
		Weighted Mean (Min−Max)	Mean (Min−Max)
Age (years)		61 (42−77)	61 (39−79)
**Characteristic**	**Values**	Weighted Percentage*	**Percentage**
Race	Caucasian	84	87
	African American	11	10
	Other	5	3
Surgery Year			
	1987−1992	11	11
	1993−2004	89	89
Biopsy Gleason Score			
	≤6	71	72
	7	25	24
	≥8	5	4
Clinical T-stage			
	T1	67	66
	T2	33	34
Pathologic T-stage			
	T2	53	
	T2+	40	
	T3	7	
Preoperative PSA	≤10	84	82
	>10−20	13	15
	>20	3	4
AUA Risk Group	Low	57	58
	Intermediate	33	34
	High	11	9

* The patients sampled in this study functionally represent the entire cohort of 2,641 patients. Weighted percentages represent the percentage of patients in the category with the respect to the full cohort.

Note: numbers may not add up to 100 due to rounding.

Quantitative gene expression was measured in primary and highest Gleason pattern tumor regions as well as adjacent normal-appearing tissue from RP specimens

### Gene identification in normal-appearing tissues adjacent to tumor tissues

**Figure 2:**
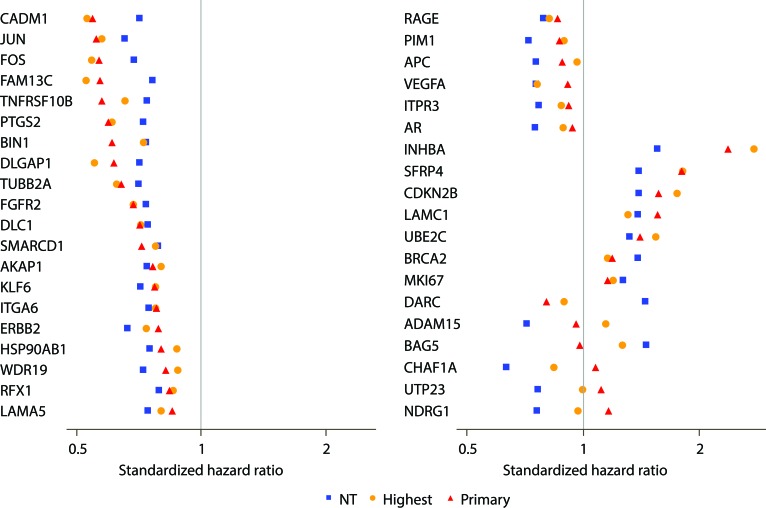
Comparison of strength of genes in predicting clinical recurrence when assessed in adjacent normal-appearing (NT) and tumor tissues (primary or highest Gleason pattern) Forest plot representing the association of each of the 39 genes found to be predictors of clinical recurrence (FDR < 20%) in both tumor and NT. Standardized hazard ratios are shown as red triangles for the primary Gleason pattern tumor, orange circles for the highest Gleason pattern tumor, and blue squares for the adjacent NT.

Of 732 candidate cancer-related genes assayed, 5 genes were excluded due to poor analytical performance, yielding 727 evaluable genes (Figure 1a). Controlling the FDR at 20%, 46 genes were predictive of cR in NT, indicating that gene expression patterns in NT adjacent to prostate tumor can predict clinical outcome in PCa. Among these 46 genes, 39 (85%) were associated with cR in tumor tissues as well (FDR < 20%) (Figure 2, Table 2).

**Table 2:** Functional groups of 39 Genes Associated (FDR < 20%) with Clinical Recurrence in NT

Androgen Signaling	Stromal Response	Cellular organization	Proliferation	Stress Response	Cell Adhesion	Chromatin remodeling and genome stability	Other
AR	INHBA	BIN1	DLC1	BAG5	ADAM15	CHAF1A	APC
ERBB2	SFRP4	FGFR2	CDKN2B	PTGS2	AKAP1	SMARCD1	BRCA2
FAM13C	LAMA5	TUBB2A	MKI67	FOS	RFX1		DARC
NDRG1	LAMC1		UBE2C	HSP90AB1	CADM1		ITPR3
WDR19				JUN	DLGAP1		RAGE
				KLF6	ITGA6		TNFRSF10B
				PIM1			UTP23
							VEGFA

The genes associated with cR in NT represent a diverse range of biological pathways: androgen signaling, stromal response, cellular organization, proliferation, cell adhesion, chromatin remodeling and genome stability, protein folding, and stress response (Table 2). Higher expression of stromal response and proliferation genes was associated with higher risk of clinical recurrence, whereas, for the androgen signaling, cellular organization, chromatin remodeling, protein folding, and stress response gene groups, reduced expression was associated with higher recurrence risk (data not shown).

The standardized HRs for these 39 genes in NT ranged from 0.6 to 0.8 for genes associated with better outcome and 1.3 to 1.6 for genes associated with worse outcome; for a majority of these genes (77%; 30/39), the association was such that down-regulated gene expression was associated with worse outcome. For 33 of these 39 genes, the association of gene expression with clinical recurrence was directionally consistent in NT and tumor, although a stronger association was usually observed in tumor compared with NT (Figure 2a).

**Figure 3:**
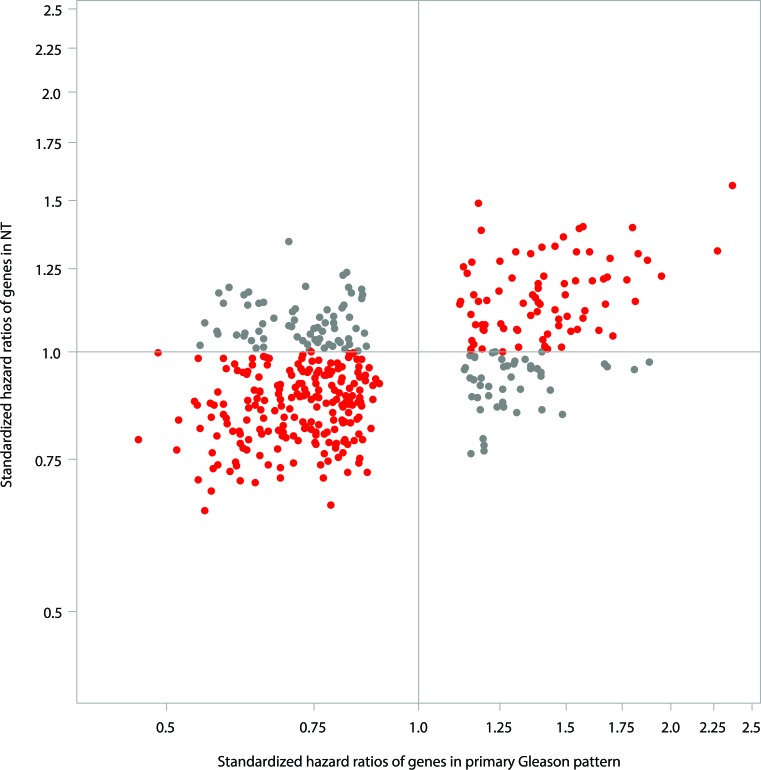
Comparison of strength of genes in predicting clinical recurrence in NT *vs*. tumor (primary Gleason pattern) for the 405 genes, which were predictive of clinical recurrence in tumor (FDR < 20%) Red dot - q-values < 0.2 in primary Gleason pattern in predicting clinical recurrence and Std. HR were in the same direction between NT and primary Gleason pattern; Gray dot - q-values < 0.2 in primary Gleason pattern in predicting clinical recurrence and Std. HR were in the opposite directions between NT and primary Gleason pattern.

The stronger association between gene expression and cR in tumor compared to NT is also supported by the observation that a large number of genes were associated with cR when analyzed in the tumor itself. A total of 405 genes were found to be associated with cR in tumor tissue (either primary or highest Gleason pattern) at an FDR at 20%. The majority of these genes (*n* = 289, 71%) demonstrated similar but weaker associations with cR in NT (Figure 3).

To exclude the possibility that aberrant gene expression patterns observed in NT were due to inadvertent contamination with tumor cells, first we imposed a physical boundary of ≥3mm to separate NT sample from tumor foci, and then we assessed the presence of T2-ERG fusions in both NT and tumor. The frequency of T2-ERG fusions was 55% in tumor specimens, consistent with other published cohorts [28, 29]. The frequency of detectable T2:ERG fusions in NT was 12% (28/225) in cases with T2-ERG positive tumors, consistent with other studies showing the presence of T2-ERG fusions in adjacent normal-appearing prostate tissue in 5-15% of cases,[28] [30] suggesting that contamination of NT with tumor cells was an uncommon event in this study.

### Assessment of the predictive value of the tumor-derived GPS in normal-appearing tissue

The tumor-derived 17-gene GPS, when assessed in NT, was significantly associated with cR (RM-corrected HR per 20 units = 1.25; 95% CI: 1.01−1.56; *P* = 0.024). For comparison, the RM-corrected HR per 20 units for the association of GPS with cR when assessed in tumor was 3.42; 95% CI: 2.33−5.63; *P* < 0.001. Among 12 informative individual GPS genes, two genes of the GPS genes were identified in NT; one stromal response gene (SFRP4) and one androgen signaling gene (FAM13C1) predicted cR in NT in univariable analysis (FDR < 20%). Inspection of the individual pathways represented by the genes that constitute GPS showed that the androgen signaling gene group (RM-corrected Std. HR = 0.92; 95% CI 0.84−1.00; *P* = 0.041) and the stromal response gene group (RM-corrected Std. HR = 1.10; 95% CI: 1.02− 1.20) in NT were each predictive of cR. Proliferation (TPX2) and cellular organization group components of the GPS were not significantly associated with cR in NT (Table 3).

**Table 3:** Univariate analyses of GPS gene groups as predictors of clinical recurrence when assessed in normalappearing adjacent tissue (NT) and tumor (primary Gleason pattern) tissue

Gene Group	Assessed in NT			Assessed in primary Gleason pattern tumor		
	**RM-Corrected Std. HR**	**95% CI**	***P*-value**	**RM-Corrected Std. HR**	**95% CI**	***P*-value**
**Androgen Signaling**	0.92	(0.84, 1.00)	0.041	0.52	(0.44, 0.61)	<0.001
**Stromal Response**	1.10	(1.02, 1.20)	0.016	1.69	(1.38, 2.07)	<0.001
**Cellular Organization**	1.00	(0.93, 1.08)	0.95	0.63	(0.52, 0.77)	<0.001
**Proliferation**	1.08	(0.99, 1.17)	0.068	1.50	(1.30, 1.73)	<0.001

## DISCUSSION

In this analysis of gene expression in 410 paired NT and tumor prostate specimens, we identified 39 genes whose expression showed an association with clinical outcome when measured in NT adjacent to prostate tumors as well as in tumor tissue. These 39 genes represent diverse biologic pathways, including androgen signaling, stromal response, cellular adhesion, and apoptosis. These results support a biologically meaningful field cancerization in PCa, demonstrating that gene expression patterns in NT adjacent to PCa can predict clinical outcome. Furthermore, the tumor-based 17-gene GPS, which was derived from tumor samples within this dataset, was associated with clinical outcome when measured in normal tissue, although the strength of association was weaker than in tumor.

Evidence for a field effect in PCa is supported by an extensive body of literature showing a wide range of cytomorphological, genetic, and epigenetic changes in NT in tumor-containing prostate glands [14]. Many studies have shown that gene expression patterns in NT adjacent to prostate tumors are altered compared to NT from non-tumor bearing glands [5, 6, 31-33]; however it is noteworthy that none of these studies showed that those expression patterns predict the clinical aggressiveness of the associated tumor. More recently, Cooper et al. have shown in prostatectomy specimens that microdissected normal tissue distant from tumor contains a high frequency of mutations that mirror those seen in the cancers, suggesting a field effect of clonal expansion involving similar biologic processes in both tumor and normal tissue [34].

Another notable finding was that in a majority (30/39) of the predictive genes identified in NT, down-regulation of expression was associated with worse clinical outcome. This observation is consistent with prior studies showing extensive DNA methylation in adjacent NT; a large number of the methylation sites involved gene promoter regions and were associated with down-regulation of gene expression [2].

Other than the Cooper study, these prior studies typically utilized fresh-frozen tumor samples rather than more conventional formalin-fixed specimens [5, 31], and often included laser capture microdissection to enrich for prostate epithelium [32, 35], a method not widely used in clinical pathology laboratories. Comparison of our study with these prior studies revealed little overlap in the identified genes, with the exception of stress-response genes such as Fos [5, 33, 36]. However, stress response genes are known to be induced by hypoxic conditions during radical prostatectomy [37-39] and, thus are not ideal biopsy-based biomarkers. Our study is the first to demonstrate an association between gene expression in histologically appearing normal tissue and clinical outcome.

In addition to discovering genes associated with outcome in prostate NT, we showed that a tumor-derived prognostic signature, the GPS, was associated with clinical recurrence when assessed in NT. Although the HR for GPS was smaller than the HR observed in the tumor tissue, this finding indicates that the score is robust to the presence of NT that may be present on prostate biopsy. The relative weakness of the association between clinical outcome and GPS measured in NT *versus* tumor is not surprising, given that the genes that comprise the GPS were selected based on their predictive value when measured in the tumor tissue itself [15]. On the other hand, the fact that GPS in NT is also predictive of clinical outcome further validates GPS as a measure of aggressive disease. Among the genes within the GPS, the strongest predictors of outcome within the NT regions were genes associated with stromal response and androgen signaling.

While this study provides evidence for a field effect in adjacent NT, the study design did not permit a determination of the extent of this field effect throughout the gland, since normal tissue was sampled from a single area at least 3 mm from the tumor. In addition, this study was restricted to the expression measurements of 727 cancer-related genes. A more comprehensive survey, utilizing next generation sequencing techniques and tissue sampling from various non-tumor regions of the tumor-bearing gland would likely reveal a larger spectrum of genetic and gene expression changes and delineate their geographic distribution within the prostate.

The identification of prognostic gene expression patterns in adjacent NT may have additional clinical value for the estimated 750,000 men screened annually for PCa who have negative prostate biopsies [40], since 25−30% of negative prostate biopsies are false negatives due to under-sampling [41]. It is conceivable that a prognostic gene expression signature could be used to predict the presence of a cancer that was not sampled on the first biopsy, as has been shown for tests based on other molecular alterations, including gene-specific methylation patterns [42] and mitochondrial DNA deletions [3]. Further study is required to determine whether the genes identified in this study could predict the presence of clinically significant PCa after an initial negative biopsy. [40] [41] [42] [3]

Our findings also raise concerns about the potential use of focal therapy, wherein only tumors that are visible on multi-parametric MRI are treated, while the remaining epithelium is spared. As observed by Cooper, focal therapy may be curative only if surrounding clonal-cell populations within morphologically normal tissue are also ablated. Further study is required to determine whether the use of these or similar markers of field cancerization might be useful in selecting patients for this form of therapy.

Strengths of our study include the use of a well-characterized clinical cohort of contemporary radical prostatectomy patients, expert pathology review, and microdissection of tumor and NT, use of a hard clinical endpoint (clinical tumor recurrence and not biochemical failure), an unbiased approach to gene selection after initial identification of genes of interest, a pre-specified analysis plan, and use of T2:ERG fusion status to rule out contamination of NT by tumor. The main weakness of our study is that the genes assessed as predictors of clinical outcome in NT were initially selected from expression studies in tumor tissue, and may not reflect the full spectrum of the molecular field effect outside the tumor.

In conclusion, gene expression profiles in NT adjacent to prostate tumor, including the previously validated GPS, predict PCa outcome. These findings suggest that there is a biologically significant field effect in primary PCa that is a marker for aggressive disease. The results have implications for understanding PCa biology, cancer detection, and the success of treatments using subtotal gland ablation.

## MATERIALS AND METHODS

### Patients and tissue specimens in the gene identification study

Stratified cohort sampling [19] was used to select 501 patients with (*n* = 127) and without (*n* = 374) clinical recurrence (distant metastases or local recurrence) using a 1:3 ratio of cases to non-cases, from 2,641 early-stage PCa patients treated by radical prostatectomy at the Cleveland Clinic from 1987−2004 (Figure 1a) [15]. Eligible patients had clinical stage T1/T2, at least one follow-up assessment, and available fixed paraffin-embedded (FPE) prostatectomy tumor tissue. Exclusion criteria included neoadjuvant or adjuvant therapy, < 5% tumor area occupied by invasive cancer cells, insufficient RNA ( < 325 ng), and poor RNA quality. Disease and vital status were determined from a prospectively maintained, institutional review board−approved, HIPAA-compliant database.

### Pathology review and tissue sampling

All RP specimens were centrally reviewed by two urologic pathologists specialized in PCa (SF and CMG) to assess primary and secondary/tertiary Gleason patterns and overall Gleason score using the 2005 International Society of Urological Pathology Consensus guidelines [20], pathologic stage, and tumor location (peripheral *versus* transition zone). For each patient, two spatially distinct tumor specimens, representing the primary Gleason pattern and the highest Gleason pattern, were manually microdissected using a microscope. A single area of NT adjacent to PCa was also microdissected for RNA extraction and analysis, while maintaining ≥ 3 mm distance from tumor (Figure 1b). Whenever possible, the NT specimen was selected adjacent to the primary Gleason pattern. Unstained FPE tumor sections (six 10-μm sections used for RT-PCR analysis plus two 5- μm top and bottom sections used for H&E staining) were prepared by Cleveland Clinic personnel and analyzed by Genomic Health (Redwood City, CA). The first and last sections were H&E stained (5-μm sections) to confirm presence or absence of tumor, and the first H&E slide was used to guide manual microdissection from intervening six identically oriented unstained sections. [15]

### TMPRSS2-ERG analysis

To exclude the possibility that aberrant gene expression patterns observed in NT were due to inadvertent contamination with tumor cells, we assessed the presence of TMPRSS2-ERG (T2-ERG) fusions, a common rearrangement in PCa, in both NT and tumor. Samples were considered T2-ERG fusion positive if the expression of either TMPRSS2-ERGa or TMPRSS2-ERGb was higher than the limit of quantitation [21, 22]. A separate construct measuring the expression of ERG was used to verify the total number of fusion-positive samples.

### Genomic prostate score (GPS) assay

GPS is a quantitative reverse transcriptase polymerase chain reaction assay (RT-PCR - TaqMan^®^, Life Technologies, Carlsbad, CA) which measures the mRNA levels of 17 genes (12 cancer-related and 5 reference genes), and provides the GPS on a scale of 0−100. The 12 cancer-related genes, which represent 4 biologic pathways including androgen signaling, stromal response, cellular organization, and proliferation, were selected based on their association with clinical outcome, including clinical recurrence, when measured in prostate tumor. The details of the development strategy and clinical validation of this assay have been previously published [15-17]. Because this paper reports the analysis of GPS assessed retrospectively in the gene identification study, GPS is defined as the combination of the 17 genes as described in the assay specifications, but does not represent the commercial-grade GPS assay.

### Statistical methods

Descriptive statistics and time to event analyses were weighted to account for the sampling nature of the original study design. The primary objective for this analysis was to identify genes whose expression in NT adjacent to PCa was associated with clinical recurrence-free interval (cRFI), defined as the time from surgery to first distant metastases or local recurrence as established by imaging or biopsy. We also evaluated the ability of GPS, which was developed in this same cohort using tumor tissue samples only, to predict cRFI when measured in NT.

For cRFI, losses to follow-up and non−PCa—related deaths prior to recurrence were censored at the time of the last observation. Univariable Cox proportional hazards (PH) regression models using weighted pseudo-partial likelihood estimators [23] with robust variance estimation developed by Gray [19] were used. The proportional hazards assumption was evaluated according to Therneau and Grambsch [24]. Storey's method [25] was used as a conservative approach to control the false discovery rate (FDR) at 20%, a control rate that is typical of discovery studies [26] [27]. Hazard ratio (HR) for GPS was calculated per 20-units, representing the difference between the average GPS of the highest 25th and lowest 25th percentiles of patients. The HR for GPS was corrected for regression to the mean to account for over-optimism in the estimate resulting from using the same cohort for discovery of the genes and development of the GPS. All statistical hypothesis tests were 2-sided and *P*-value < 0.05 was considered significant. Analyses were performed using SAS version 9.4 and JMP version 11.0.0 (SAS Institute Inc., Cary, NC).

### Statement of translational relevance

Efforts to design prognostic molecular diagnostic tests for prostate cancer are made challenging by 1) the inherent heterogeneity and multifocality of most prostate tumors, and by 2) the variable admixture of malignant and benign cells in the sampled tissue. A sizeable body of evidence indicates a variety of genomic alterations are shared by prostate tumor cells and adjacent normal-appearing tissue, indicating the presence of a generalized field effect in tumor-bearing prostate glands. However the bulk of published data has not indicated that the molecular changes in NT could predict the aggressiveness of the nearby cancer. Here we show that gene expression patterns which are predictive of long-term outcomes, such as clinical recurrence after radical prostatectomy, can be observed in NT. This finding suggests that prognostic tumor-based gene expression signatures may capture, at least in part, this biologically relevant field effect.
